# Complete Resection without Any Ostomies by Laparoscopic Extended Surgery for Locally Advanced T4 Sigmoid Colon Cancer Invading the Urinary Bladder and Ureter

**DOI:** 10.1155/2019/9598183

**Published:** 2019-12-21

**Authors:** Atsushi Ogura, Tsukasa Aritake, Satoru Kawai, Shigeki Yamamoto, Kenji Takagi, Kiyotaka Kawai, Takashi Maeda, Ryutaro Kobayashi, Natsuki Nagano, Satoaki Kamiya

**Affiliations:** ^1^Department of Surgery, Tsushima City Hospital, 3-73, Tachibana Town, Tsushima City, Aichi, Japan; ^2^Department of Urology, Tsushima City Hospital, 3-73, Tachibana Town, Tsushima City, Aichi, Japan

## Abstract

The feasibility and safety of laparoscopic surgery for locally advanced colorectal cancer remain controversial due to the high rate of incomplete resection and conversion to open surgery. Especially for T4 colorectal cancer, laparoscopic techniques are still demanding mainly because of the difficulty in distinguishing between inflammation and tumor involvement, which often lead surgeons to do overtreatment in surgery. We believe laparoscopic magnified and multidirectional approach might be useful for pathologically complete resection and minimizing an unnecessary extended surgery for these cases. A 49-year-old man was diagnosed with locally advanced T4 sigmoid colon cancer invading the urinary bladder and ureter. We performed laparoscopic anterior resection with en bloc resection of the urinary bladder and the left ureter. Total operative time was 462 min, and the estimated blood loss was 50 ml. This patient was discharged on the 28th day after surgery without any ostomies and urinary functional disorders. The magnified view by laparoscopic techniques from multiple directions would enable surgeons to set surgical landmarks for another approach, which is the key for safe and feasible laparoscopic surgery in patients with locally advanced T4 colorectal cancer.

## 1. Introduction

Laparoscopic surgery has been widely used in the recent decades and can be an acceptable option as a therapeutic approach for colorectal cancer with almost similar perioperative and survival outcomes as that achieved with open surgery [1–5]. However, the feasibility and safety of laparoscopic surgery for locally advanced colorectal cancer, particular T4 cancer, were reported in some retrospective-fashioned articles [6, 7] and remain controversial due to the high rate of incomplete resection and conversion to open surgery, which resulted in the poor short- and long-term outcome [1].

Sigmoid colon cancer in certain cases becomes a large mass involving the bladder and occupies the upper pelvic space in front of the promontory. Considering both the curability of surgery and the quality of life after surgery, it is very difficult to make a decision regarding the best surgical plan between open and laparoscopic surgery or pelvic exenteration and local resection among other such approaches for these patients. We believe that the magnified view afforded by laparoscopic techniques from multiple directions could be especially helpful in narrow surgical fields such as the pelvis and enables surgeons to set surgical landmarks for another approach, which is the key for safe laparoscopic surgery in patients with locally advanced colorectal cancer.

Here, we report our experience of a case of locally advanced sigmoid colon cancer invading the urinary bladder and ureter wherein complete resection without any ostomies by laparoscopic surgery was performed.

## 2. Case Presentation

**Figure 1:**
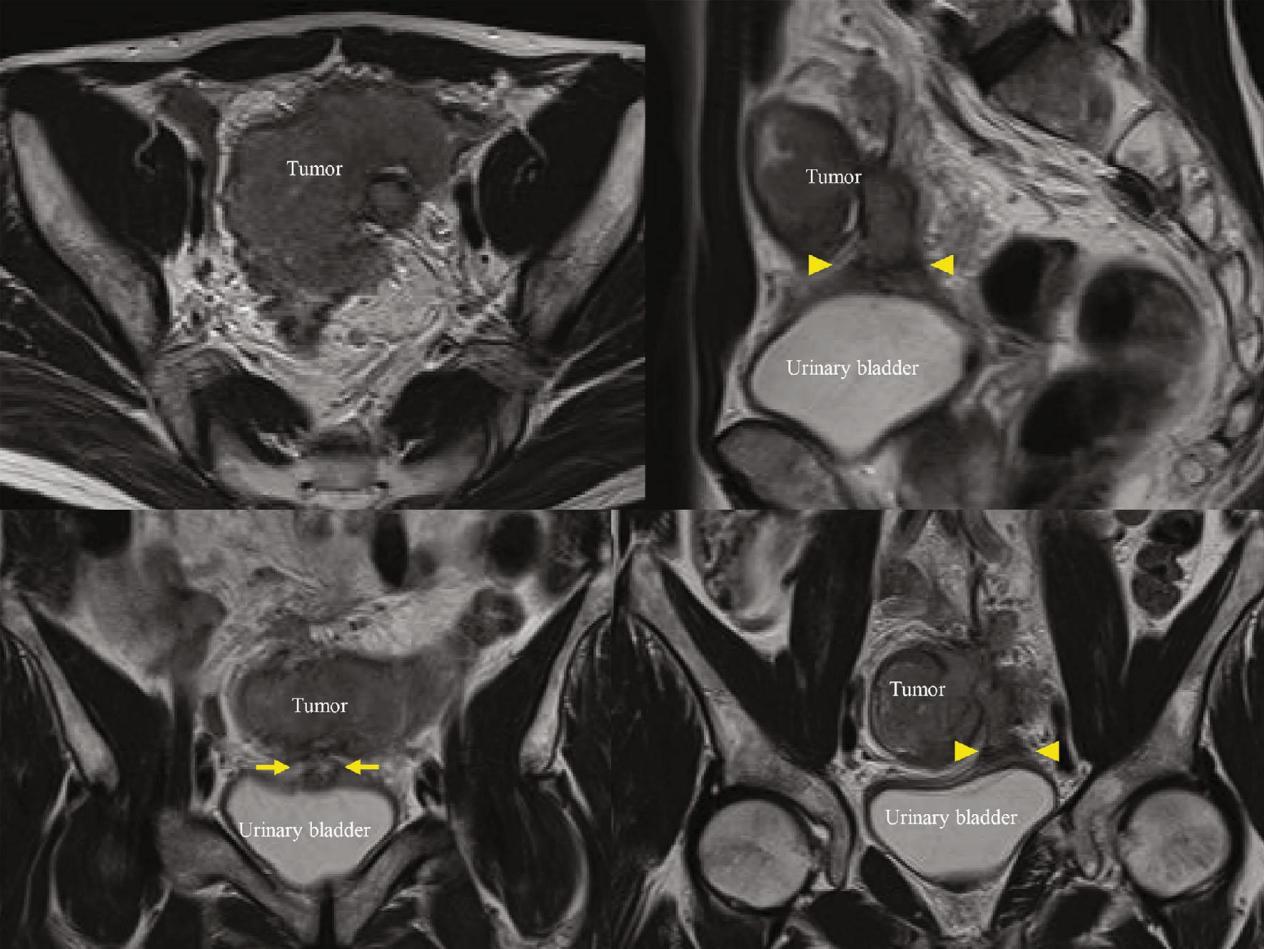
Magnetic resonance images show a large tumor directly invading the urinary bladder (arrowhead) and an abscess between the tumor and urinary bladder (arrow).

**Figure 2:**
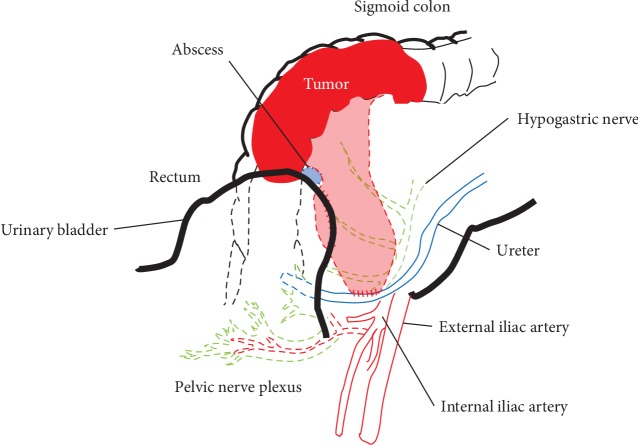
Schema of the tumor and peripheral organs.

A 49-year-old man was referred to our hospital for lower abdominal pain. Colonoscopy showed a circumferential tumor in the sigmoid colon, and histopathological examination revealed moderately differentiated adenocarcinoma. Enhanced computed tomography (CT) and magnetic resonance imaging (MRI) showed a large tumor directly invading into the urinary bladder and the left ureter. Besides, an abscess was formed between the tumor and the urinary bladder (Figures 1 and 2). Cystoscopy showed no definitive tumor exposed in the mucous membrane, and urine cytology was also negative for cancer. Preoperative staging was cT4bN0M0 cStage IIC locally advanced sigmoid colon cancer. Neoadjuvant therapy was considered; however, because the patient had continuous lower abdominal pain with a high C-reactive protein level of 6.1 mg/L, we decided against neoadjuvant therapy. We then planned laparoscopic anterior resection with en bloc partial resection of the urinary bladder and left ureter. For a safe surgical margin and to avoid bleeding from the internal iliac vessels, en bloc resection of the hypogastric and pelvic nerve plexus was also planned preoperatively.

The patient was placed in the lithotomy position under general and epidural anesthesia. The following ports were placed: a 12 mm port at the umbilicus for a scope, a 12 mm port at the lower right quadrant, and 5 mm ports at the upper right and left abdominal quadrants.

**Figure 3:**
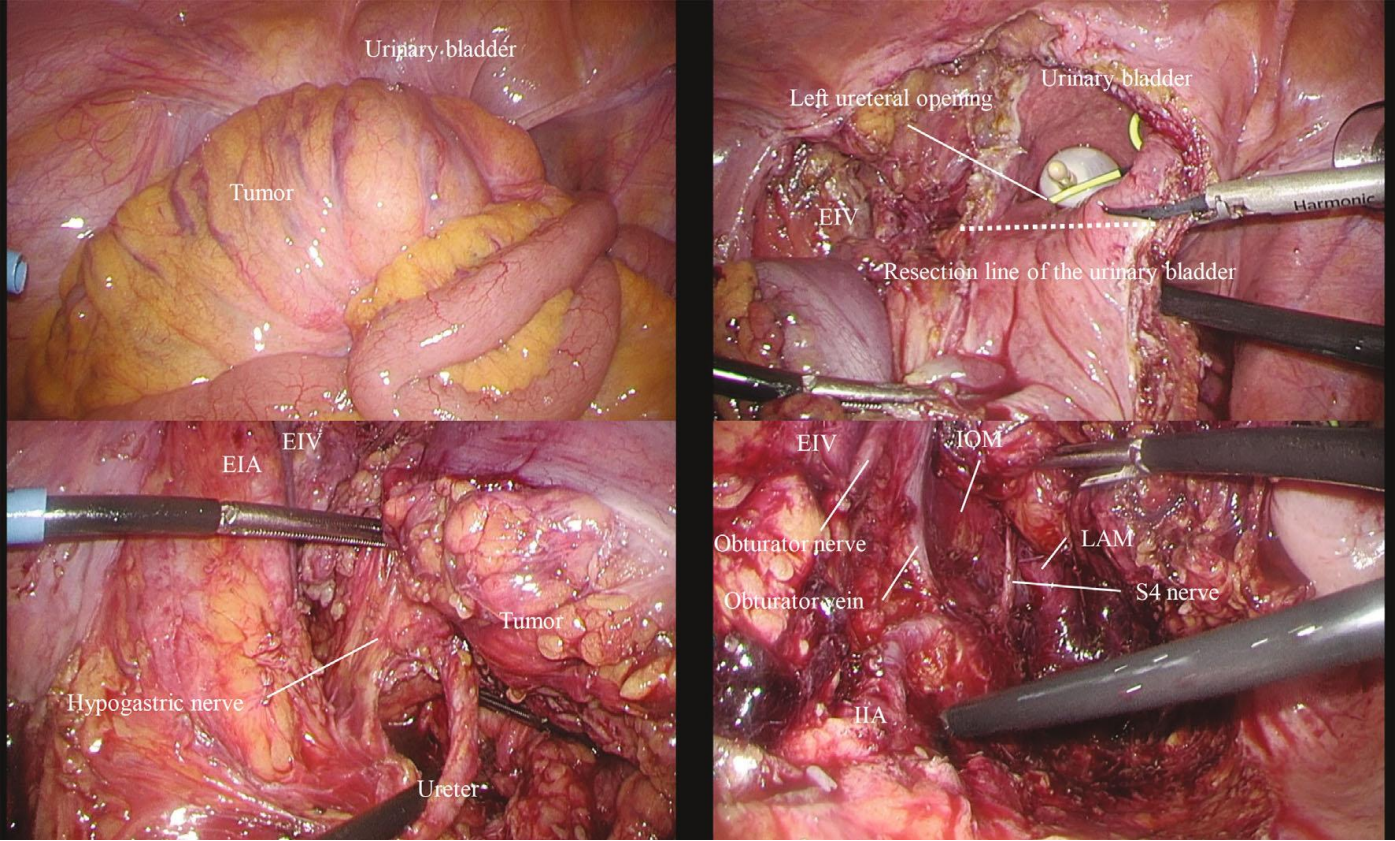
Laparoscopic views before and after the extended surgery with en bloc resection of the urinary bladder, left ureter, and left lateral lymph nodes. EIV: external iliac vein; EIA: external iliac artery; IOM: internal obturator muscle; LAM: levator ani muscle; IIA: internal iliac artery.

First, the sigmoid colon was mobilized medially-to-laterally and the root of the inferior mesenteric artery was divided. Subsequently, we mobilized the rectum to the bottom of the pelvic floor on the right and posterior sides while completely preserving the right nerve plexus; this point was used as the surgical landmark on the posterior side (Figure 3).

Second, the external iliac vein was exposed and the obturator nerve and vessels were preserved. The left ureter was mobilized and divided at the oral side of the tumor. The left internal iliac artery was exposed and the umbilical artery and inferior vesical vessels were divided. At the distal side of the lateral pelvic area, we achieved the landmark on the posterior side in front of the levator ani muscle with en bloc resection of the hypogastric nerve and pelvic nerve plexus; this point was used as the surgical landmark on the lateral side (Figure 3).

Next, the urinary bladder was opened at the proximal side with a sufficient surgical margin and the left ureteral opening with the ureteral stent was recognized. We resected the distal wall of the urinary bladder by connecting the line from the right side of the rectum to the surgical landmark on the lateral side (Figure 3).

**Figure 4:**
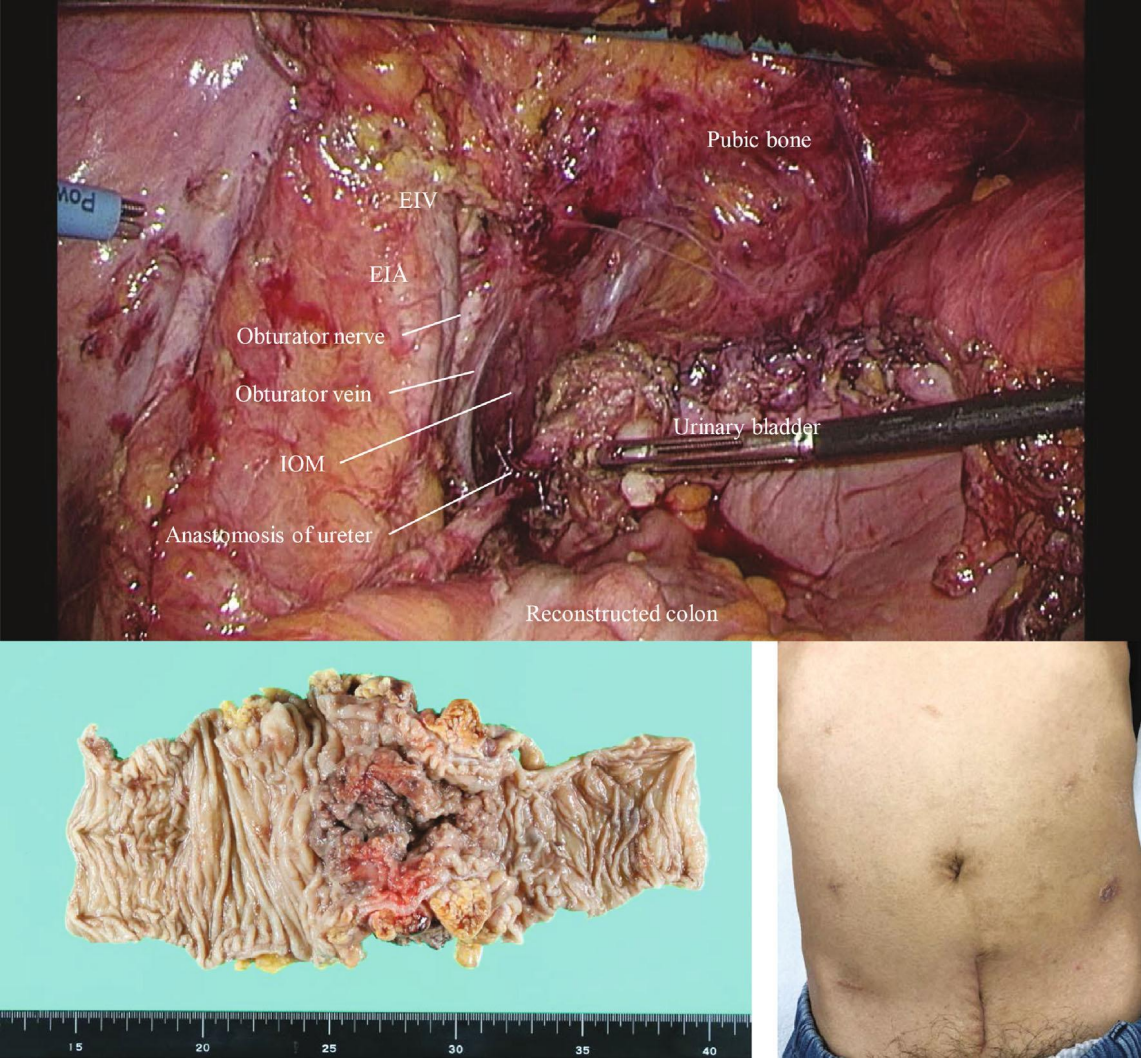
Laparoscopic view after the reconstruction, the photo of the resected specimen, and the photo of the abdomen 1 month after the surgery. EIV: external iliac vein; EIA: external iliac artery; IOM: internal obturator muscle.

Finally, the mesorectum was resected circumferentially on the distal side and transected. The tumor was removed from an 8 cm long lower abdominal incision. Intraoperative pathology was negative for cancer at the distal wall of the urinary bladder. The reconstruction of the left ureter and the urinary bladder was performed under direct vision, and the reconstruction of the rectum was also performed laparoscopically (Figure 4).

The total operative time was 462 min, and the estimated blood loss was 50 ml. The pathological results revealed that the tumor involved the muscle layer of the urinary bladder and the outer membrane of the left ureter; however, the results were negative for cancer at the margin (Figure 4). No lymph node metastasis was detected. The pathological diagnosis was pT4bN0M0 pStage IIC. Minor leakage at the ureteral anastomosis and small bowel obstruction were resolved by conservative treatment. This patient was discharged on the 28th day after surgery without any ostomies and urinary functional disorders and once urine output has been established: larger than 200 ml.

## 3. Discussion

We experienced a unique case of locally advanced sigmoid colon cancer invading the urinary bladder and ureter that was managed with complete resection without any ostomies by laparoscopic surgery. There are some previous reports concerning pelvic exenteration for locally advanced rectal cancer invading the urinary bladder [8–10]. Moreover, if a tumor penetrates the bladder wall, pelvic exenteration becomes inevitable to achieve curative resection. As in the present case wherein the tumor had invaded up to the muscle layer of the urinary bladder, an additional optional procedure may be selected such as partial resection of the urinary bladder and left ureter. However, this selection should be made with caution because it is often difficult to distinguish an inflammation due to tumor involvement intraoperatively.

The magnified view facilitated by laparoscopy from multiple directions appears to be suitable for setting surgical landmarks for another approach. In the present case, mobilizations of the posterior and lateral sides of the tumor were performed in advance to set surgical landmarks for safe resection of the urinary bladder and left ureter. A large mass in the narrow pelvis can make it difficult to set surgical landmarks in open surgery. We thus believe that setting surgical landmarks during laparoscopic surgery could be the key to achieve a safe and curative resection for T4 colorectal cancer leading not only to less blood loss and postoperative pain but also high quality of life without any ostomies. However, the technique described here should be performed by laparoscopic surgeons with extensive experience in advanced colorectal cancer surgery because true invasion was only a half of cT4b colorectal cancer and it was difficult to distinguish between inflammation and tumor involvement [11].

In conclusion, setting a surgical landmark during laparoscopic surgery using the magnified view from multiple directions could potentially enable complete resection without any ostomies in selected patients with T4 sigmoid colon cancer invading the urinary bladder and ureter.
